# The Expression of Alamandine Receptor MrgD in Clear Cell Renal Cell Carcinoma Is Associated with a Worse Prognosis and Unfavorable Response to Antiangiogenic Therapy

**DOI:** 10.3390/ijms25031499

**Published:** 2024-01-25

**Authors:** Gorka Larrinaga, Asier Valdivia, Inés Arrieta-Aguirre, Jon Danel Solano-Iturri, Aitziber Ugalde-Olano, Ana Loizaga-Iriarte, Aida Santos-Martín, Amparo Pérez-Fernández, Javier C. Angulo, José I. López

**Affiliations:** 1 Department of Nursing, Faculty of Medicine and Nursing, University of the Basque Country (UPV/EHU), 48940 Leioa, Spain; ines.arrieta@ehu.eus; 2 Department of Physiology, Faculty of Medicine and Nursing, University of the Basque Country (UPV/EHU), 48940 Leioa, Spain; 3 Biobizkaia Health Research Institute, 48903 Barakaldo, Spain; jondanel.solanoiturri@osakidetza.eus (J.D.S.-I.); aitziber.ugaldeolano@osakidetza.eus (A.U.-O.); ana.loizagairiarte@osakidetza.eus (A.L.-I.); aida.santosmartin@osakidetza.eus (A.S.-M.); amparo.perezfernandez@osakidetza.eus (A.P.-F.); joseignacio.lopez@biocrucesbizkaia.org (J.I.L.); 4 Department of Cellular Biology and Histology, Faculty of Medicine and Nursing, University of the Basque Country (UPV/EHU), 48940 Leioa, Spain; asier.valdivia@ehu.eus; 5 Department of Pathology, Cruces University Hospital, 48903 Barakaldo, Spain; 6 Department of Pathology, Basurto University Hospital, 48903 Barakaldo, Spain; 7 Department of Urology, Basurto University Hospital, University of the Basque Country (UPV/EHU), 48013 Bilbao, Spain; 8 Clinical Department, Faculty of Medical Sciences, European University of Madrid, 28905 Getafe, Spain; javier.angulo@universidadeuropea.es; 9 Department of Urology, University Hospital of Getafe, 28907 Madrid, Spain

**Keywords:** renal cell carcinoma, MrgD receptor, alamandine, renin–angiotensin system, prognosis

## Abstract

Renal cell carcinoma (RCC) ranks among the most prevalent malignancies in Western countries, marked by its notable heterogeneity, which contributes to an unpredictable clinical trajectory. The insufficiency of dependable biomarkers adds complexity to assessing this tumor progression. Imbalances of several components of the intrarenal renin–angiotensin system (iRAS) significantly impact patient prognoses and responses to first-line immunotherapies. In this study, we analyzed the immunohistochemical expression of the Mas-related G-protein-coupled receptor D (MrgD), which recognizes the novel RAS peptide alamandine (ALA), in a series of 87 clear cell renal cell (CCRCCs), 19 papillary (PRCC), 7 chromophobe (ChRCC) renal cell carcinomas, and 11 renal oncocytomas (RO). MrgD was expressed in all the renal tumor subtypes, with a higher mean staining intensity in the PRCCs, ChRCCs, and ROs. A high expression of MrgD at the tumor center and at the infiltrative front of CCRCC tissues was significantly associated with a high histological grade, large tumor diameter, local invasion, and locoregional node and distant metastasis. Patients with worse 5-year cancer-specific survival and a poorer response to antiangiogenic tyrosine-kinase inhibitors (TKIs) showed higher MrgD expression at the center of their primary tumors. These findings suggest a possible role of MrgD in renal carcinogenetic processes. Further studies are necessary to unveil its potential as a novel biomarker for CCRCC prognosis and response to frontline therapies.

## 1. Introduction

Renal cell carcinoma (RCC) ranks among the most frequently diagnosed malignancies in Western countries [1,2,3]. Clear cell renal cell carcinoma (CCRCC) constitutes the predominant histological subtype, encompassing approximately 75–80% of cases, followed by papillary renal cell carcinoma (PRCC) at 10–15% and chromophobe renal cell carcinoma (ChRCC) at 5% [4,5]. The renal oncocytoma (RO) is a benign tumor with an incidence of 5% among all renal tumors [4,5]. CCRCC and PRCC are widely accepted to originate primarily from the proximal convoluted tubule of the nephron, whereas ChRCC and RO appear to stem from the intercalated cells of the distal nephron [4,5]. CCRCC, the most aggressive RCC variant, manifests with 30% of cases being metastatic at diagnosis and an additional 30% of patients with localized disease progressing to metastatic stages [4,5].

Recent studies have identified a close correlation between specific genomic signatures and the clinical aggressiveness of RCC [6], and a more accessible delineation of alterations linked to tumor behavior and clinical outcomes is imperative for effective advancements in managing RCC patients. In recent years, the exploration of novel prognostic biomarkers and therapeutic modalities has gained momentum [5,7]. Thus, the study of the components of the renin–angiotensin system (RAS) in renal tumors and the potential utility of drugs targeting this peptidergic system have emerged as promising RCC research avenues [8,9,10].

The RAS is an endocrine peptidergic system crucial in kidney and cardiovascular physiology [11]. In addition to this circulating system, local RASs exist, such as the intrarenal RAS (iRAS), which regulate long-term biological processes through paracrine and autocrine mechanisms [8,11,12]. In physiological conditions, the angiotensin-converting enzyme (ACE)–angiotensin-II (Ang-II)–Ang-II receptor (AT1R) axis locally induces cell growth, angiogenesis, and tissue repair, whereas the ACE2–angiotensin 1–7–(Ang 1–7)–MAS receptor (MASR) pathway counterbalances these signals [11,13]. Imbalances in favor of the first axis lead to cell proliferation, inflammation, and fibrosis of the kidney [11,13]. The discovery of these pathophysiological phenomena has enhanced our understanding of the effectiveness of ACE inhibitors (ACEis) and AT1R blockers (ARBs) in arresting the progression of non-neoplastic kidney diseases such as diabetic nephropathy [11,13] and broadened the focus on investigating the iRAS in renal carcinogenesis [14,15,16,17,18].

Alamandine (ALA) is a recently discovered new member of the RAS [19]. The binding of this peptide to the Mas-related G-protein-coupled receptor D (MrgD) exerts similar physiological actions to the Ang 1–7–MASR pathway, leading to antifibrotic and antiproliferative effects in local contexts [19,20,21]. Nevertheless, the existing literature on the expression of MrgD in tumor tissues and the role of the ALA–MrgD axis in cancer development and progression remains notably limited [22,23,24].

Given the observed imbalances of various components of the main axes of the iRAS in RCC, which significantly impact patient prognoses [14,25,26,27,28], imbalances could be expected in the MrgD expression within this tumor. Hence, this pilot study aimed to analyze the immunohistochemical (IHC) expression of this receptor in a series of 124 renal tumors. We analyzed both the tumor center and the infiltration front to explore potential heterogeneity in MrgD expression in RCC. Considering the prominence of CCRCC as the most prevalent subtype among RCCs [4,5], our study also investigated the association between MrgD and tumor progression and its impact on the prognosis of CCRCC patients.

## 2. Results

### 2.1. The Expression of the MrgD Receptor Exhibits Variability Based on the Specific Subtype of Renal Tumor

**Figure 1:**
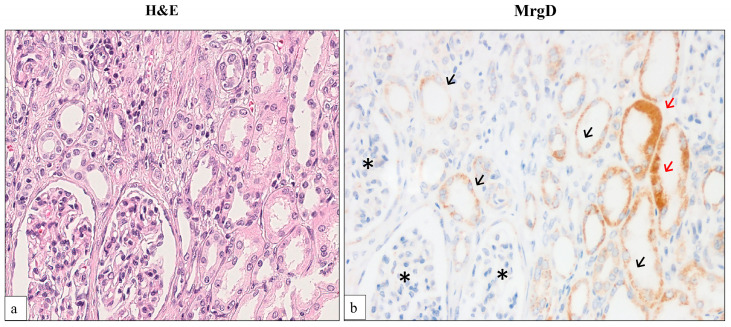
Distribution of MrgD immunostaining in non-tumor renal tissue. Hematoxylin–eosin and MrgD immunostaining of the non-tumor kidney. (**a**) Renal cortex includes glomeruli (asterisk), proximal convoluted tubules (red arrow), and tubules of the distal nephron (black arrow). (**b**) Unlike glomeruli, tubular cells express MrgD with a granular cytoplasmic or membranous staining pattern. Whereas proximal tubules are intensely stained with MrgD antibody, distal nephron tubules display weak/negative staining. Original magnification, ×400.

We first tested the MrgD receptor expression at the non-tumor part of the kidney. This protein was expressed with a varied intensity at the cytoplasm and the membrane of cells along the tubules of the nephron. Thus, the staining was moderate/intense at proximal tubules, whereas it was weak/negative at tubules from the distal nephron and negative at glomeruli (Figure 1).

**Figure 2:**
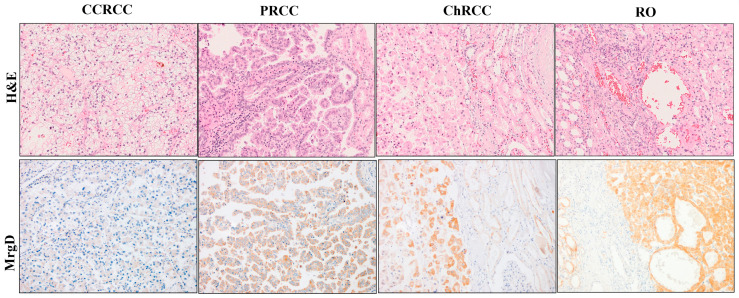
MrgD immunostaining pattern in kidney tumors. MrgD immunostaining pattern in different renal tumor subtypes [clear cell renal cell carcinoma (CCRCC), papillary renal cell carcinoma (PRCC), chromophobe renal cell carcinoma (ChRCC), and renal oncocytoma (RO)]. Original magnification, ×250. H&E: Hematoxylin–eosin.

**Figure 3:**
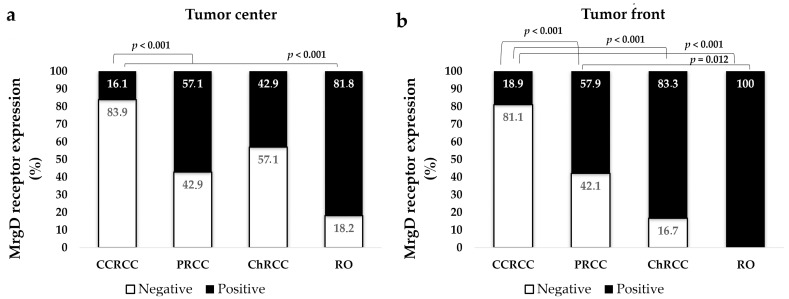
MrgD staining intensity at the tumor center (**a**) and front (**b**). Staining intensity was grouped as negative and positive. PRCCs, ChRCCs, and ROs showed significantly higher MrgD expression than CCRCCs. Chi-square test was used for data analysis.

Based on observations in the non-tumor renal tissue, the staining pattern of the renal tumors was classified into two groups: tumors with moderate or intense cytoplasmic and membranous staining were considered to be positive cases, whereas tumors not stained with the MrgD receptor and those showing only weak staining were grouped as negative. Significant differences in the staining pattern existed between tumor subtypes. Thus, the expression of MrgD was negative in more than 80% of CCRCCs and positive in more than half of PRCCs, 60–80% of ChRCCs, and 80–100% of ROs (Figure 2 and Figure 3).

We additionally assessed differences in MrgD receptor expression between the tumor center and the infiltrating front within each tumor subtype. No significant differences were observed between these locations within CCRCCs (Chi-square test, *p* = 0.637), PRCCs (*p* = 0.962), ChRCCs (*p* = 0.135), or ROs (*p* = 0.138) (Figure 3).

### 2.2. The Expression of MrgD in CCRCC Varies Depending on the Tumor’s Aggressiveness

The data obtained from the CCRCC tumors were categorized based on pathological parameters closely linked to tumor aggressiveness, such as WHO/ISUP histological grade, size, local invasion (pT), the presence or absence of affected lymph nodes (N) and metastases (M), and tumor necrosis. The association between MrgD and clinical variables such as patients’ sex, age, and 5-year cancer-specific survival (CSS) was also analyzed. Data from patients with advanced CCRCC were also stratified depending on their response to antiangiogenic therapy, classified by the MASS and RECIST criteria.

The initial statistical analysis revealed no correlation between MrgD expression at the tumor center or front and the patients’ age (tumor center: Spearman rho, r = 0.099, *p* = 0.361; tumor front: r = 0.145, *p* = 0.219) or gender (tumor center: chi-s, *p* = 0.679; tumor front, *p* = 0.568).

#### 2.2.1. MrgD Expression Is Higher in High-Grade CCRCCs

**Figure 4:**
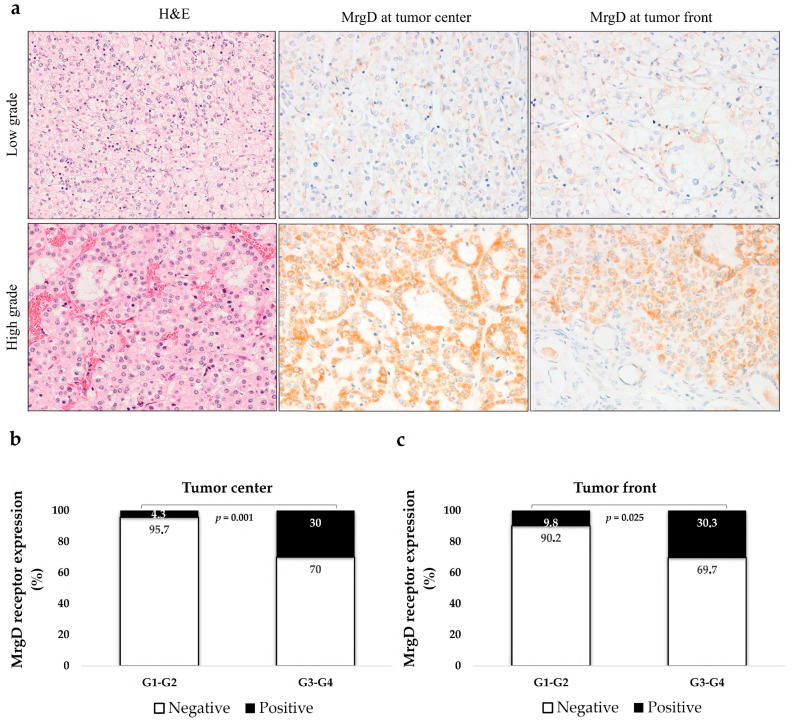
MrgD expression according to tumor grade. Immunostaining at the tumor center and front of low- and high-grade CCRCCs (**a**). Original magnification, ×400. H&E: Hematoxylin–eosin. MrgD staining intensity both at the center (**b**) and front (**c**) of tumors was grouped as negative and positive. MrgD staining was more intense in high-grade CCRCCs. Chi-square test was used for data analysis.

We categorized the cases into low (G1–G2) and high histological grade (G3–G4). High-grade CCRCCs showed significantly elevated MrgD staining compared to low-grade tumors, at both the tumor center and the infiltrating front (Figure 4).

#### 2.2.2. MrgD Staining Is More Intense in Large (>7 cm) Tumors

**Figure 5:**
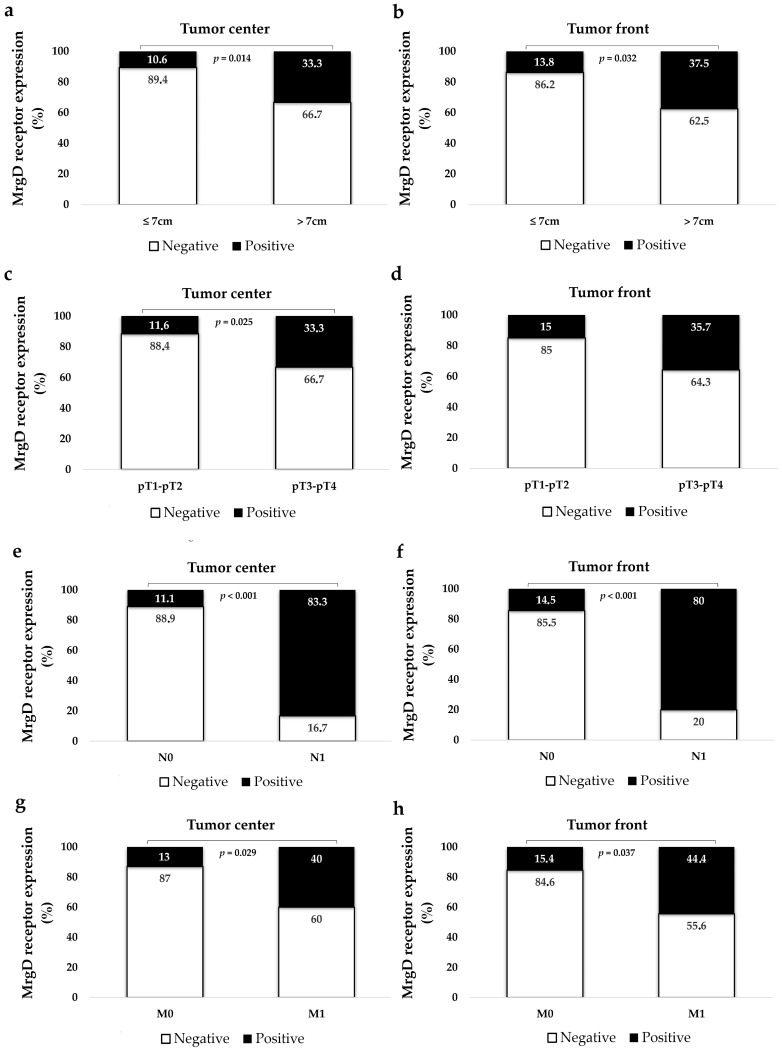

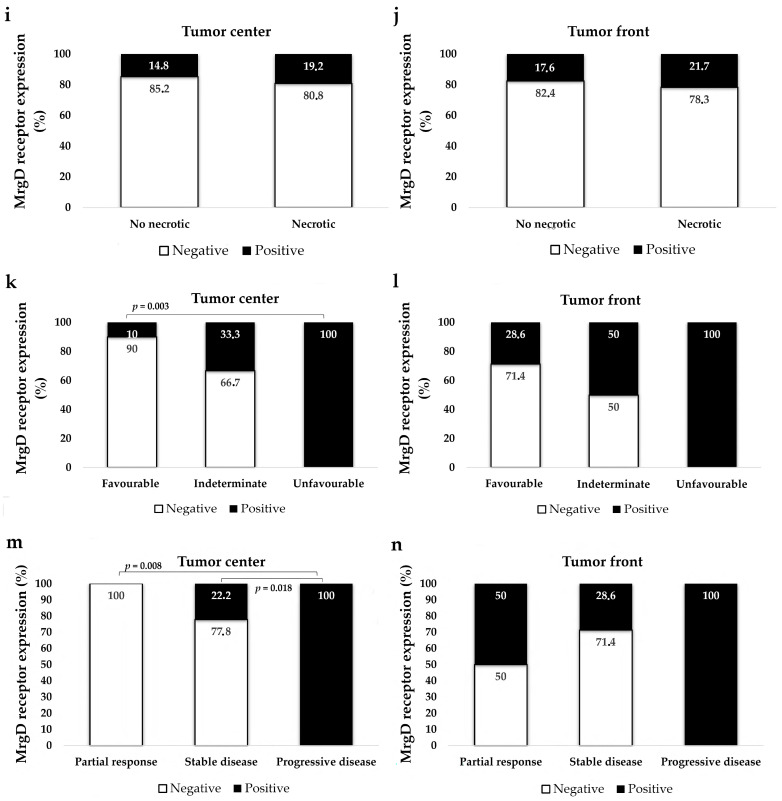
Immunohistochemical staining of MrgD based on CCRCC tumor size (**a**,**b**), local invasion (pT; **c**,**d**), lymph node (N; **e**,**f**), distant metastasis (M; **g**,**h**), and tumor necrosis (**i**,**j**). Results were also categorized depending on patients’ response to therapy with TKIs, according to MASS (**k**,**l**) and RECIST (**m**,**n**) criteria. MrgD staining intensity was grouped as negative and positive. Chi-square test was used for data analysis. N0: No lymph node metastasis; N1: lymph node metastasis; M0: no distant metastasis; and M1: distant metastasis.

CCRCCs were classified into two groups: tumors of 7 cm or smaller and those larger than 7 cm. The MrgD staining exhibited increased intensity in larger CCRCCs compared to smaller ones. This difference was statistically significant both at the tumor center and at the periphery (Figure 5).

#### 2.2.3. MrgD Expression Is Higher in Non-Organ-Confined CCRCCs

The tumors were classified into two groups: organ-confined (pT1–pT2) vs. non-organ-confined tumors (pT3–pT4). The MrgD staining was more intense in non-organ-confined cases, which was significant at the tumor center (Figure 5).

#### 2.2.4. MrgD Expression Is Significantly Higher in Metastatic CCRCCs

Tumors that invaded locoregional lymph nodes (N1) exhibited increased MrgD staining intensity at both tumor regions compared to those without lymph node invasion (N0). Likewise, MrgD expression was elevated in CCRCCs with distant synchronous metastasis (M1) in contrast to tumors without. However, CCRCCs with necrotic areas displayed no significant differences in MrgD expression when compared with non-necrotic cases (Figure 5).

#### 2.2.5. MrgD Expression Is Associated with Worse Cancer-Specific Survival (CSS)

**Figure 6:**
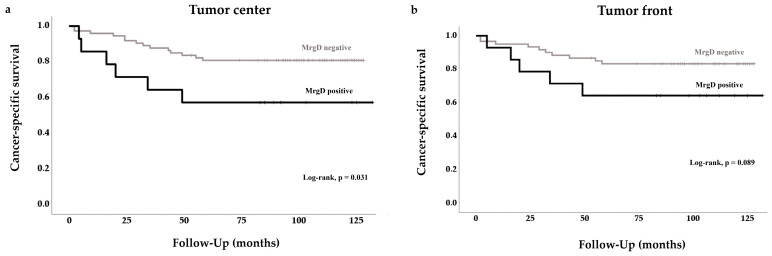
Kaplan–Meier curves for cancer-specific survival according to immunohistochemical staining of MrgD. Patients with negative expression are compared to those with positive expression: (**a**), tumor center (log-rank, *p* = 0.031); (**b**), tumor front (*p* = 0.089).

MrgD positivity at the tumor center was associated with poorer 5-year CSS in CCRCC patients (Figure 6). A similar trend was observed at the tumor front, although it did not reach statistical significance (Figure 6).

A univariate analysis was conducted to assess the individual correlation between MrgD and pathological variables with patients’ survival. The univariate Cox regression model demonstrated that grouped grade (G1/2 vs. G3/4), tumor diameter, pT stage (organ-confined vs. non-organ-confined), lymph node invasion (pN), distant metastasis (pM), and MrgD expression correlated with a poorer 5-year CSS (Supplementary Table S1). The variables that reached statistical significance (*p* < 0.05) in the univariate analysis were included in the multivariate Cox regression model. To prevent mathematical bias, tumor diameter was omitted from the analysis because local invasion (pT) encompasses this variable. The logistic model resulting from a backward Wald stepwise elimination of variables with a 0.1 stay criterion identified pT (confined vs. non-confined), pN (negative vs. positive), and pM (negative vs. positive) as independent prognostic factors for CSS (Table 1). Grade and MrgD expression were not identified as independent predictors in this model.

#### 2.2.6. MrgD Expression Is Associated with Worse Response to Antiangiogenic Therapy

We categorized the data from the IHC study of MrgD based on the MASS and RECIST criteria for responses to TKIs in patients with advanced CCRCC. A gradual increase in MrgD expression was observed at the tumor center among patients with favorable, indeterminate, and unfavorable responses (Chi-s, *p* = 0.013; Figure 5). A similar significant gradient was noted in the same tumor area among patients with a partial response, those with stable disease, and non-responder patients with progressive disease (Chi-s, *p* = 0.013; Figure 5). Categorized by the MASS criteria, the expression of MrgD at the tumor margins showed a similar gradual expression, although it was not statistically significant (Chi-s, *p* = 0.2).

## 3. Discussion

MrgD belongs to the large family of Mas-related G-protein-coupled receptors [20]. Discovered at the beginning of this century [29], limited information is available regarding its expression and function in most tissues. Despite its identification as a component of the RAS [19], few data exist on its expression in the kidney beyond studies conducted in cell lines [20] and mRNA detection in human renal tissue [22]. In this study, we present the IHC characterization of MrgD in both non-tumor and tumor tissues from the excised kidneys of RCC patients. As reported for other components of the iRAS [10,11], we detected this receptor at the membrane and cytoplasm within tubular cells from both proximal and distal nephrons.

Knowing that these are the topographic origins of the main renal tumors [4,5], the presence of this receptor could be expected in CCRCC, PRCC, ChRCC, and RO tissues. Indeed, it was expressed in tumor cells from both the center and the invading front of the four renal tumor subtypes. Interestingly, the mean expression of MrgD in CCRCC was significantly lower than in the rest of the analyzed tumor subtypes. Although this could suggest a less relevant role of this novel RAS receptor in the most frequent and aggressive kidney tumor [4,5], primary CCRCCs with a worse prognosis significantly increased its expression. The MrgD staining was more intense in tumors with a high histological grade, larger size, lymph node involvement, and synchronous distant metastasis. Furthermore, patients’ 5-year CSS was worse when this receptor was highly expressed at the center of the primary tumor. Apart from its prognostic implications, our observations revealed an association between MrgD expression and a poorer response to TKIs, antiangiogenic drugs that represent the first therapeutic line for advanced CCRCC together with immune checkpoint inhibitors (ICIs) [30].

The association between imbalances of the iRAS and renal tumor aggressiveness remains an ongoing area of investigation [10]. The most significant advancements have been documented concerning the classical axis of the RAS. Preclinical studies revealed an upregulation of AT1R in tumor cells [14], as well as an increased expression of ACE in tumor vessels [25,26], in CCRCCs with a poorer prognosis. Furthermore, cancer-associated fibroblasts (CAFs) and tumor-infiltrating immune cells such as myeloid-derived suppressor cells (MDSCs) express the AT1 receptor and ACE [31,32,33]. These cells can create immunosuppressive microenvironments conducive to tumor invasion [34], which is enhanced by the stimulation of the Ang-II–AT1R signaling pathway and inhibited by the use of ARBs in combination with ICIs [31,32,33]. Aligning with this, retrospective studies have indicated that CCRCC patients receiving ACEis and ARBs exhibit a better response to TKIs and ICIs [9,15,16,17,18]. This accumulated evidence suggests a significant pro-tumor role of the classical axis of the RAS in renal cancer [10], which impacts directly on cancer immunity [33].

The role of non-classical pathways of RAS in renal carcinogenesis remains more controversial. Khanna et al. [35] reported that Ang1–7 inhibits tumor growth and that high ACE2 mRNA levels are associated with a better survival, which corroborates the theory of counterbalancing effects of the ACE2–Ang1–7–MasR axis in other proliferative disorders [10,12]. However, other authors have demonstrated pro-invasive effects of Ang1–7 in RCC cell lines and xenografts [36,37] and a higher ACE2 protein expression in high-grade CCRCCs [26].

The ALA–MrgD pathway has also been described as a counter-regulator of the classical axis [20]. For instance, ALA inhibits fibrosis in the heart [19], liver [38], and lungs [39] by suppressing TGF-β1-induced fibroblast activation [21]. However, initial studies on cancer have yielded conflicting results. Thus, the binding of ALA to MrgD in pancreatic cancer cell lines induces an antiproliferative effect by inhibiting the BRAF–MKK–ERK and PI3K–AKT pathways and activating FoxO1 [24]. Conversely, in non-small-cell lung cancer, ALA stimulates cell growth and MrgD is expressed more intensely in more aggressive tumors [22,23], which aligns with our results for CCRCC.

We also observed inter-tumor heterogeneity when examining four renal tumor subtypes, which agrees with our previous studies on other RAS components [26,27]. Within tumors originating in the proximal tubule, the mean expression of MrgD in PRCC was significantly higher than that in CCRCCs. In addition, its expression in tumors from the distal nephron (ChRCC and RO) was higher than that in proximal-nephron-derived ones. Furthermore, a higher expression of this receptor was observed in RO, a benign tumor. Taken together, these findings suggest a heterogeneous role of the ALA–MrgD pathway, depending on the tumor type and subtype.

Concerning CCRCC, it is also crucial to consider the existing high genotypic and phenotypic intratumoral heterogeneity (ITH) [40] when interpreting the divergent results described within non-canonical RAS pathways [26,34,35,36]. For instance, the antagonistic effects of Ang1–7–Mas signaling could be associated with the use of cell lines and xenograft models derived from distinct tumor cell subpopulations, as suggested by Sobczuk et al. [36]. Therefore, to determine whether this ITH is reflected in the spatial distribution of the MrgD receptor, we examined its expression at the tumor center and margins. Consistent with a previous report where we studied the (pro)renin receptor [27], MrgD staining displayed uniformity across both tumor areas. This discovery implies that the inherent heterogeneity of CCRCC might not impact its IHC analysis, favoring the potential use of MrgD as a prognostic biomarker.

In summary, this pilot study demonstrates the MrgD expressions in the four more prevalent renal tumors. Furthermore, the results in CCRCCs show that the expression of this receptor is increased at both the center and at the invasive front of tumors with a worse prognosis and unfavorable response to TKIs. These outcomes must be validated in a larger CCRCC series to unveil the true potential of this protein as a biomarker for prognosis and response to frontline therapies. Furthermore, studies in cell lines and animal models will be pivotal to shed light on the role of this novel ALA–MrgD pathway in RCC hallmarks. Additionally, investigating the possible involvement of MrgD in CCRCC through RAS-independent mechanisms will be important, given its affinity for other endogenous ligands but also its constitutive activity, which has been detected in renal (HEK293) [41] and tumor (HeLa) cell lines [42]. All these efforts will aim to comprehend the potential of MrgD as a novel therapeutic target in RCC.

## 4. Materials and Methods

### 4.1. Patients

A total of 113 RCCs surgically removed at Basurto University Hospital between 2012 and 2016 were collected for the study: 87 CCRCCs (mean age: 61.7 years, 60 male and 27 female patients) (Table 2), 19 PRCCs (mean age: 53.5 years, 15 male and 4 female patients), 7 ChRCCs (mean age: 63.9 years, 6 male and 1 female patient), and 11 ROs (mean age: 63.4 years, 4 males and 7 females).

Samples from the tumor center and the infiltrating front were included in tissue microarrays (TMAs) for further IHC analyses. Two samples of the non-tumor renal tissue were also included in each TMA.

### 4.2. MASS and RECIST Response Criteria for Patients with Metastatic CCRCC

In the studied series, 23 patients experienced metastasis (10 synchronous and 13 metachronous), 16 of whom were treated with antiangiogenic drugs, specifically TKIs. As this series falls within the TKI era, immune checkpoint inhibitors were only administered to a limited number of patients following the progression on TKI treatment.

To evaluate the response assessment to systemic therapy, both the MASS (Morphology, Attenuation, Size, and Structure) [43] and RECIST (Response Evaluation Criteria in Solid Tumors) criteria [44] were employed. According to the MASS criteria, patients with advanced CCRCC were categorized into three groups, depending on favorable (n = 10), indeterminate (n = 3), or unfavorable (n = 3) response to treatment. Based on RECIST, patients were stratified as having a partial response (n = 4), stable disease (n = 9), or progressive disease (n = 3).

### 4.3. Immunohistochemistry

The immunostaining of formalin-fixed and paraffin-embedded tumor tissues was performed with a rabbit polyclonal antibody specific for MrgD (ref. HPA031346; Sigma-Aldrich, St. Louis, MO, USA) at a 1/50 dilution. The antibody’s specificity had been previously validated [45]. The immunostaining process followed standard procedures using an automated immunostainer (Dako Autostainer Plus, Dako-Agilent, Glostrup, Denmark). Antigen retrieval was conducted in a low-pH buffer (K8005, Dako) at 95 °C for 20 min. The samples were incubated with the primary antibody at room temperature for 50 min, then washed and treated with a secondary anti-rabbit antibody (K8021, Dako) for 20 min. The EnVision-Flex detection system, combined with an HRP enzyme-labeled polymer (SM802, Dako), was used. A positive reaction was visualized using diaminobenzidine (DAB) solution (DM827, Dako) followed by counterstaining with hematoxylin (K8008, Dako).

The slides were examined under light microscopy for staining assessment. Two independent observers evaluated the slides, and in cases of discrepancies, samples underwent re-evaluation to reach a final consensus.

Each TMA contained two cores of non-tumor renal tissue serving as controls. We defined the MrgD staining observed in proximal tubules as moderate or intense, while the staining in the distal nephrons and glomeruli was categorized as weak and negative, respectively (see Figure 1). In the same way, we defined MrgD staining in a tumor core as weak, moderate, or intense when at least 10% of the tumor cells in that core showed weak, moderate, or intense staining, as in the control samples, and we defined the staining as negative when it did not reach 10% of the core [46]. Finally, the staining pattern was categorized in two groups: tumors with moderate or intense cytoplasmic and membranous staining were considered to be positive cases, whereas tumors with weak or negative staining were grouped as negative.

### 4.4. Statistical Analysis

The statistical analysis was performed with SPSS^®^ 28.0 (IBM Corp., Armonk, NY, USA).

We applied the Spearman Rho test to assess whether the IHC data obtained from tumor tissues were correlated to patients’ age (which is a quantitative variable). The association between qualitative variables such as the categorical MrGD expression (negative vs. positive), patients’ sex, the pathological variables of the CCRCC patients (described in Table 2), and response to TKIs, was tested using the Chi-square (χ^2^) test. To evaluate the association between MrgD and the CSS of the CCRCC patients, Kaplan–Meier curves and log-rank tests were carried out. Finally, to evaluate the independent effects of this protein or pathological variables on CSS, we used univariate and multivariate analyses (Cox regression model together with the backward Wald method).

## Figures and Tables

**Table 1:** Predictive model (Cox regression) for cancer-specific survival prediction by MrgD and pathological variables in CCRCC patients. Selected independent variables were MrgD (negative vs. positive) at tumor center and front, and pathological variables such as WHO/ISUP grade (low- vs. high-grade), confined (pT1–pT2) vs. non-confined (pT3–pT4), lymph node invasion (pN, no vs. yes), and distant metastases (pM, no vs. yes). ExpB with confidence interval (CI, inferior and superior) is also included. Variables resulting from the backward Wald stepwise method are highlighted in bold.

		Tumor Center				Tumor Front			
Cancer-Specific Survival	Variables	p	ExpB	Inf	Sup	p	ExpB	Inf	Sup
Multiple Cox regression	MrgD	0.959	0.96	0.24	3.76	0.714	0.77	0.19	3.07
	Grade	0.367	1.76	0.51	6.00	0.228	2.51	0.56	11.2
	pT	0.053	2.53	0.99	6.51	0.270	1.89	0.61	5.86
	pN	0.197	2.88	0.58	14.4	0.170	3.10	0.62	15.6
	pM	0.003	5.55	1.77	17.4	0.005	5.83	1.71	19.9
Final step of Wald method	**pT**	**0.028**	2.82	1.12	7.10	-	-	-	-
	pN	0.069	3.08	0.91	10.4	-	-	-	-
	**pM**	**0.001**	6.97	2.48	19.6	**0.001**	13.43	4.75	37.9

**Table 2:** Pathological and clinical characteristics of CCRCC patients.

CCRCC Patients (n = 87)	Average (Range)
**Age** (range)	61.7 (36–82)
**Sex** (male/female)	60/27
**Follow-up** (months)	88.6 (2–132)
**Survival** (n)	**(n)**
Alive	62
Dead of disease	20
Dead by other causes	5
**Diameter** (n)	
≤7 cm	67
>7 cm	21
**WHO/ISUP grade** (n)	
Low (G1–G2)	47
High (G3–G4)	40
**Necrosis** (n)	
No	61
Yes	26
**Local invasion (pT)** (n)	
Organ-confined (pT1–pT2)	69
Not confined (pT3–pT4)	18
**Lymph node invasion (N)** (n)	
No	81
Yes	6
**Synchronous metastasis (M)** (n)	
No	77
Yes	10

## Data Availability

Anonymized datasets used and/or analyzed during the current study are available from the corresponding author upon reasonable request.

## Supplementary Materials

The following supporting information can be downloaded at: https://www.mdpi.com/article/10.3390/ijms25031499/s1.
