# Supplementary material for: The Expression of Alamandine Receptor MrgD in Clear Cell Renal Cell Carcinoma Is Associated with a Worse Prognosis and Unfavorable Response to Antiangiogenic Therapy
Source: Int J Mol Sci. 2024 Jan 25;25(3):1499. doi: 10.3390/ijms25031499 (PMC10855800; doi:10.3390/ijms25031499)
Supplement: Supplementary file 1 [file ijms-25-01499-s001.zip › ijms-2793824-supplementary.pdf]

Supplementary Table S1. Univariate Cox regression analysis for cancer-specific survival (CSS) prediction in CCRCC patients. Selected independent variables were MrgD expression at both locations of the tumor and pathological variables. ExpB with confidence interval (CI, inferior and superior) is also included. Significant results ($p<0.05$) are highlighted in bold.

Pathological Variables	p =	ExpB	Inf	Sup
**MrgD (tumour centre)**	**0.039**	2.74	1.05	7.13
**MrgD (tumour front)**	0.104	2.44	0.83	7.14
**Histological Grade** (G1-G2 vs G3-G4)	**0.005**	4.33	1.57	11.9
**Diameter** (≤ >7 cm)	**0.001**	4.32	1.79	10.4
**Local Invasion** (pT1-2 vs pT3-4)	**0.001**	4.41	1.82	10.7
**Lymph node invasion** (No/Yes)	**0.001**	11.2	3.92	32.0
**Distant Metastasis** (No/Yes)	**0.001**	12.1	4.84	30.4
**Necrosis** (No/Yes)	0.115	2.03	0.84	4.91
